# Bivalence Mn_5_O_8_ with hydroxylated interphase for high-voltage aqueous sodium-ion storage

**DOI:** 10.1038/ncomms13370

**Published:** 2016-11-15

**Authors:** Xiaoqiang Shan, Daniel S. Charles, Yinkai Lei, Ruimin Qiao, Guofeng Wang, Wanli Yang, Mikhail Feygenson, Dong Su, Xiaowei Teng

**Affiliations:** 1 Department of Chemical Engineering, University of New Hampshire, Durham, New Hampshire 03824, USA; 2 Department of Mechanical Engineering and Materials Science, University of Pittsburgh, Pittsburgh, Pennsylvania 15261, USA; 3 Advanced Light Source, Lawrence Berkeley National Laboratory, Berkeley, California 94720, USA; 4 Chemical and Engineering Materials Division, Spallation Neutron Source, Oak Ridge National Laboratory, Oak Ridge, Tennessee 37831, USA; 5 Center for Functional Nanomaterials, Brookhaven National Laboratory, Upton, New York 11973, USA

## Abstract

Aqueous electrochemical energy storage devices have attracted significant attention owing to their high safety, low cost and environmental friendliness. However, their applications have been limited by a narrow potential window (∼1.23 V), beyond which the hydrogen and oxygen evolution reactions occur. Here we report the formation of layered Mn_5_O_8_ pseudocapacitor electrode material with a well-ordered hydroxylated interphase. A symmetric full cell using such electrodes demonstrates a stable potential window of 3.0 V in an aqueous electrolyte, as well as high energy and power performance, nearly 100% coulombic efficiency and 85% energy efficiency after 25,000 charge–discharge cycles. The interplay between hydroxylated interphase on the surface and the unique bivalence structure of Mn_5_O_8_ suppresses the gas evolution reactions, offers a two-electron charge transfer via Mn^2+^/Mn^4+^ redox couple, and provides facile pathway for Na-ion transport via intra-/inter-layer defects of Mn_5_O_8_.

## Abstract (web summary)

Rechargeable aqueous electrochemical energy storage is a promising technology but suffers from a narrow potential window. Here the authors report a surface hydroxylated Mn_5_O_8_ pseudocapacitor electrode with bivalence structure that expands the potential window to deliver high energy and power performance.

Rechargeable aqueous electrochemical energy storage (EES) devices, especially ones using earth-abundant and non-toxic materials, have shown great promise for many applications owing to their high safety, low cost and environmental friendliness^1^. However, the thermodynamically stable potential window for aqueous EES has an intrinsic limit of 1.23 V, beyond which the hydrogen evolution reaction (HER) and oxygen evolution reaction (OER) occur. The evolved gas from electrolyte decomposition also severely deteriorates the electrode. A potential window of 1.23 V is too narrow for storage devices to achieve high energy and power performance.

Extensive efforts have been made to break this limit on the potential window while retaining the benefits of aqueous energy storage systems. One approach is to adjust the pH of the electrolyte to suppress HER and/or OER^1^. An aqueous battery of 2.8 V was reported by using basic electrolyte in the anode compartment (to decrease HER potential following Nernst equation) and acidic electrolyte in the cathode compartment (to increase OER potential)^2^. A Li-ion-conductive membrane was therefore needed to separate two compartments with the drastic pH difference. Another approach to suppress gas evolution is through utilization of solid–electrolyte interphase (SEI). Such interphase is ion-conductive and but not electron-conductive, protecting the electrode (for example, Li anode) from direct contact with water^3^. Recently, Suo *et al*. reported a water-in-salt electrolyte, by dissolving concentrated Li-bis(trifluoromethane sulfonyl)imide salt in water. This electrolyte system introduces a desirable SEI that enables an aqueous Li-ion full cell operation at 2.3 V for more than 1,000 cycles^4^. Although these excellent works open up the opportunities for improving the potential window, the intrinsic limitation of the ionic conductivity in Li-ion-based aqueous systems has hindered high-rate performance of the cell, especially for the pseudocapacitive storage.

Here we report a high-rate high-voltage aqueous Na-ion full cell system based on a surface hydroxylated Mn_5_O_8_ electrode. As probed by synchrotron-based surface-sensitive soft X-ray spectroscopy (sXAS), an ice-like surface hydroxyl layer is formed after cycling. Density functional theory (DFT) calculations show that the interplay between the hydroxylated interphase and the unique bivalence (Mn^2+^_2_Mn^4+^_3_O_8_) layered structure suppresses the HER and OER with a stable potential window of 3.0 V. The system exhibits a two-electron charge transfer via Mn^2+^/Mn^4+^ redox couple, and provides facile pathway for Na-ion transport at high rate. The 3.0 V aqueous symmetric full cell exhibits a high energy density, 23 Wh kg^−1^ at a rate of 550 C, with nearly 100% coulombic efficiency and 85% energy efficiency after 25,000 charge–discharge cycles.

## Results

### Synthesis and characterizations of Mn_5_O_8_ nanoparticles

**Figure 1:**
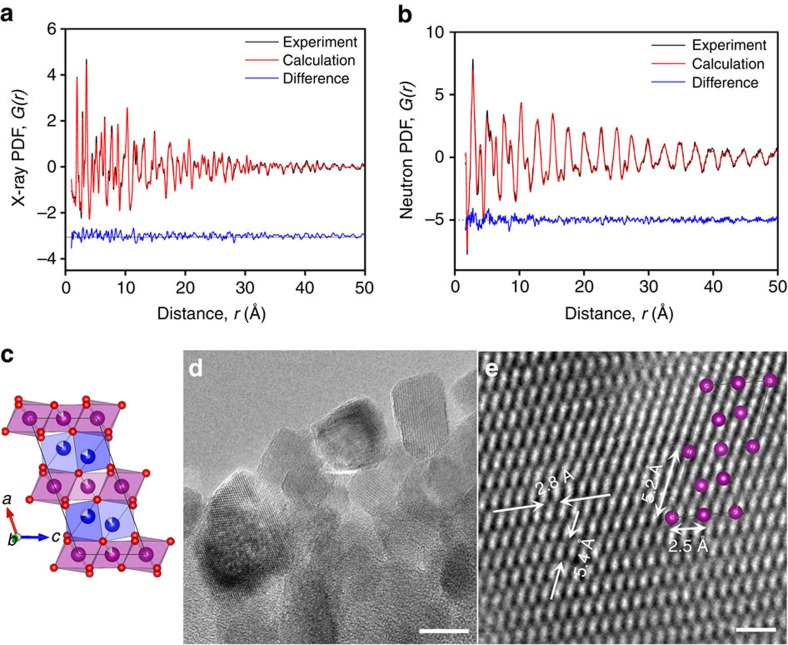
Structure characterizations of Mn_5_O_8_ nanoparticles. (**a**) X-ray and (**b**) neutron PDF analyses of Mn_5_O_8_ nanoparticles. (**c**) Lattice structure of Mn_5_O_8_ (purple: Mn^4+^; blue: Mn^2+^; red: O; white: probability of unoccupied site) obtained from PDF fitting; (**d**) TEM and (**e**) STEM images of Mn_5_O_8_ nanoparticles (inset showed Mn_5_O_8_ lattice (Mn only) along <010> direction). Scale bars, 20 nm (**d**) and 1 nm (**e**), respectively.

Mn_5_O_8_ is the only bivalent manganese oxide that has a layered structure^5^. Although Mn_5_O_8_ has abundant interlayer and intralayer defects for facile ionic transport, its capability for energy storage has rarely been reported. In this work, Mn_5_O_8_ nanoparticles were synthesized by heating Mn_3_O_4_ nanoparticles at 270 °C for 2 h in the open air. X-ray and neutron pair distribution function (PDF) analyses, shown in Fig. 1a,b, point to the formation of monoclinic Mn_5_O_8_ (Supplementary Tables 1 and 2), which consists of two-dimensional octahedral sheets of [Mn_3_^4+^ O_8_] in the *bc* plane separated by Mn^2+^ layers, giving a compositional formula of Mn^2+^_2_Mn^4+^_3_O_8_. Half of the Mn^4+^ sites in the main octahedral sheets are not fully occupied, above and below these vacant sites are Mn^2+^ sites (Fig. 1c). Apart from the major component Mn_5_O_8_, a small amount of Mn_3_O_4_ is present as seen in both the X-ray diffraction (Supplementary Fig. 1) and PDF analyses. Transmission electron microscopy (TEM) and scanning TEM showed that Mn_5_O_8_ particles have an average size of 19 nm and possess a highly crystalline monoclinic structure (Fig. 1d,e)^5^. X-ray photoelectron spectroscopy (XPS) was used to analyse the surface of Mn_5_O_8_ nanoparticles, where only manganese, oxygen, carbon and trace sodium signals can be detected without other surface residuals from synthesis and processing (Supplementary Fig. 2). XPS data show that Mn^2+^ and Mn^4+^ valences in both 2p and 3s chemical states. The XPS spectra are fitted to Mn 3 s doublets, which are split by 5.7 eV (Mn^2+^) and 4.5 eV (Mn^4+^), respectively. The integrated intensity ratio of Mn^2+^ and Mn^4+^ is calculated to be around 1: 2, close to that of theoretical value from Mn_5_O_8_ at near surface.

### Electrochemical studies of Mn_5_O_8_

**Figure 2:**
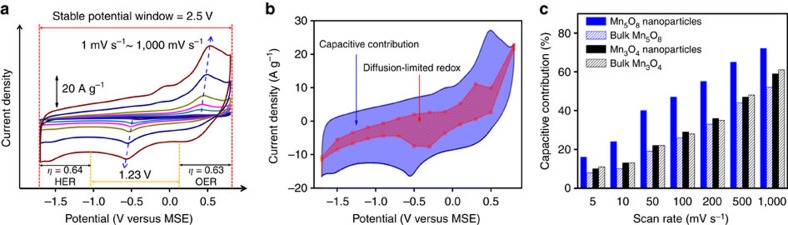
Electrochemical measurements of Mn_5_O_8_ electrodes in a half-cell. (**a**) CVs show a stable potential window of 2.5 V and large overpotential (*η*) towards HER and OER. (**b**) Electro-kinetics study of CV at 500 mV s^−1^. The contributions from capacitive process (blue) and diffusion-limited redox process (red) are shaded. (**c**) Capacitive contribution to the total charge stored at various scan rates in Mn_5_O_8_ and Mn_3_O_4_ nanoparticles and bulk materials.

Figure 2a shows the cyclic voltammetry (CV) curves of the Mn_5_O_8_-based electrodes. The Mn_5_O_8_ material was mixed with carbon black at mass ratio of 7: 3. The CV measurements were conducted using a three-electrode half-cell using a 0.1 M Na_2_SO_4_ electrolyte and a mercury sulfate electrode (MSE) as reference electrode. The CVs of carbon black show Mn_5_O_8_ material is the major contributor to the overall capacitance (Supplementary Fig. 3). The Mn_5_O_8_ nanoparticles show a wide potential window between −1.7 V (corresponding to overpotential of 0.64 V towards HER) and 0.8 V (corresponding to overpotential of 0.63 V towards OER), demonstrating the high resistance to gas evolution reactions. To our knowledge, this is the first demonstration for a stable 2.5 V potential window in an aqueous Na-ion half-cell. Moreover, the resistive capability of the Mn_5_O_8_ electrode towards HER and OER can also be demonstrated by analyzing the reaction kinetics of HER and OER using TAFEL plots (Supplementary Fig. 4). The results show that Mn_5_O_8_ has sluggish HER and OER kinetics, demonstrated by high TAFEL slopes at various scan rates, compared with various commercial Mn_3_O_4_ and Co_3_O_4_ nanopowders (Supplementary Fig. 5).

Figure 2a also shows nearly reversible current–voltage curves with discernable redox peaks from 1 to 1,000 mV s^−1^, suggesting mixed contributions from pseudocapacitive and redox processes. There are noticeable peak shifts (blue arrows) with scan rates and peak separation between anodic and cathodic scans. The peak separation increases with scan rates but in a considerably less extent compared with battery materials. The increased peak separation is typically attributed to a higher overpotential that is required to transport the ions at faster rates. Therefore, the less-distinct peak separation here indicates that our system has the potential for high-power capability with facile Na-ion transport^6^. In addition to neutral electrolyte, CVs of Mn_5_O_8_ electrodes were also conducted in Na_2_SO_4_-based electrolytes with different pH values, showing very similar behaviour in the CVs and gravimetric capacitances at the various scan rates (Supplementary Fig. 6).

Current–voltage relationship provides insight into the charge-storage mechanism. Assuming that the current (*i*) obeys the power law relationship with scan rate (*v*) at a given potential, and can be expressed as a combination of surface-controlled capacitive effects (*i*_1_=*k*_1_*v*) and diffusion-controlled Na-ion intercalation (*i*_2_=*k*_2_*v*^½^) (ref. 7):


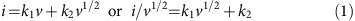


Plotting *i/v*^½^ versus *v*^½^ curves at a given potential, allows for the calculation of *k*_1_ and *k*_2_. Thus, the contributions from capacitive charge and diffusion-limited redox charge during the CV cycling are extracted quantitatively (Fig. 2b and Supplementary Figs 7 and 8). Figure 2c compares the capacitive contribution of Mn_5_O_8_ nanoparticles with Mn_5_O_8_ and Mn_3_O_4_ bulk materials, as well as Mn_3_O_4_ nanoparticles that are similar in size (18 nm) to the Mn_5_O_8_ nanoparticles. Mn_3_O_4_ is chosen since it has a bivalent structure (Mn^2+^Mn^3+^_2_O_4_) analogous to that of Mn_5_O_8_ (Mn^2+^_2_Mn^4+^_3_O_8_). Figure 2c shows that Mn_5_O_8_ nanoparticles have a greater contribution from capacitive storage compared with other materials. The results suggest that Mn_5_O_8_ nanoparticles behave as a pseudocapacitive material due to high ratio of capacitive contribution for the charge stored, as well as its unique layered structure for facile Na-ion transport.

**Figure 3:**
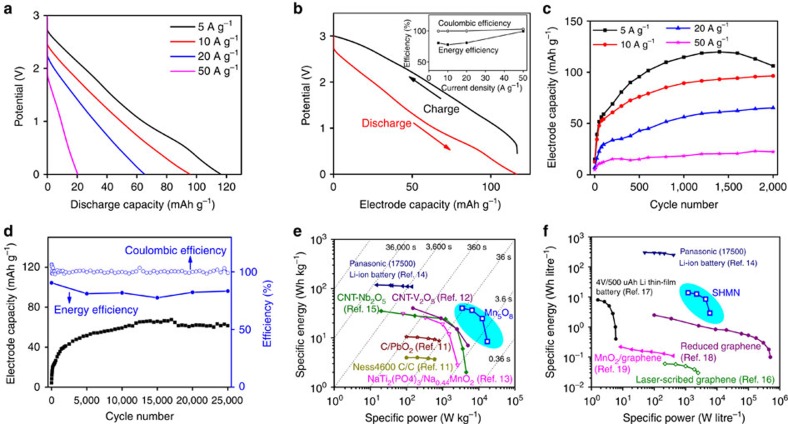
Electrochemical tests of Mn_5_O_8_symmetric cells. (**a**) Discharge capacity of Mn_5_O_8_ electrode as a function of current density; (**b**) electrode capacity during charge and discharge processes at 5 A g^−1^ (inset: energy and coloumbic efficiencies from 5 to 50 A g^−1^). (**c**) Electrode capacity as a function of cycle; (**d**) Electrode capacity and coloumbic efficiencies as functions of cycle at 20 A g^−1^. (**e**) Gravimetric and (**f**) volumetric energy and power densities of Mn_5_O_8_ cell compared with other aqueous (open symbols) and non-aqueous (solid symbols) devices. (**e**) Reported to the mass of electrode materials except Panasonic (17,500) Li-ion battery and Ness4600 C/C. (**f**) Reported to the volume of whole device. A packaging factor of 0.4 was used for Mn_5_O_8_ cell since its volumetric energy and power densities are calculated based on the volume of electrode material. The results are obtained after 2,000 charge–discharge cycles unless specified otherwise.

Symmetric full cells were assembled to evaluate the electrochemical performance of Mn_5_O_8_. Each electrode was prepared by depositing Mn_5_O_8_ (5 mg) and carbon black (1.25 mg) on carbon paper current collector (1.77 cm^2^), yielding a thickness of ∼80 μm. With 1 M Na_2_SO_4_ electrolyte, Mn_5_O_8_ cell displays a stable potential window of 3.0 V after 2,000 charge and discharge cycles, the largest stable potential window ever reported for an aqueous storage device. Nearly linear potential-capacity curves, characteristic of a pseudocapacitive response, are observed (Fig. 3a). When current densities increase from 5 to 50 A g^−1^, discharge times evolve from ∼42 s (corresponding to a rate of 85.7 C) to 0.75 s (a rate of 4,800 C), and electrode capacities change from 116 to 20 mAh g^−1^ accordingly. Figure 3b shows charge and discharge potential-capacity curves at various current densities (Supplementary Fig. 9). The discharge curve is lower than charge curve, reflecting the charge and energy losses during cycling. Coloumbic and energy efficiencies, the ratio of the amount of charge and energy that are taken from the device versus the amount that was stored during each cycle, are shown in Fig. 3b inset after 2,000 cycles. When current density increases from 5 to 50 A g^−1^, coloumbic efficiency of Mn_5_O_8_ electrode remains nearly 100%, and energy efficiency increases from 80 to 97%. Figure 3c shows good cycling stability of Mn_5_O_8_ full cells at a 3.0 V potential window from 5 to 50 A g^−1^. At 20 A g^−1^, the Mn_5_O_8_ cell shows excellent coloumbic efficiency (∼100%) and energy efficiency (∼85%), and a plausible electrode capacity of 61 mAh g^−1^ after 25,000 cycles (Fig. 3d). High coulombic and energy efficiencies, good linearity between potential and electrode capacity, as well as superior cycling stability, all suggest that Mn_5_O_8_ acts as a stable pseudocapacitive material at high rate.

Low-rate performance of Mn_5_O_8_ cell was also studied. At low current densities, electrode capacity fades rapidly (Supplementary Fig. 10). This result indicates Mn_5_O_8_ acts closer to a batteries-like electrode materials at low-rate, during which storage capacity is contributed significantly from diffusion-limited redox process. This is confirmed by non-linear voltage-capacity curves at low rate, exhibiting voltage plateaus (Supplementary Fig. 11), concurrent with the results obtained from CV measurements in half-cell showing that diffusion-limited redox process contributed predominately to the overall charge stored at low scan rates (Fig. 2c). The fact, that Mn_5_O_8_ electrode capacities show an optimal performance at a current density of 5 A g^−1^ and fade remarkably at the low current densities (for example, 0.5 and 1 A g^−1^) in the prolonged cycles, could also be attributed to a parasitic reaction from either hydrogen or oxygen evolution occurring at low rates. The resulting gas evolution could deteriorate the Mn_5_O_8_ electrode and cause the loss of capacity at prolonged cycles. It has also been pointed out that though electrode material contains Mn_3_O_4_ impurity phase (<20%), it shows very sluggish electro-kinetics of HER and OER as shown in TAFEL plots and slopes (Supplementary Figs 4 and 5), indicating high overpotential towards HER and OER at off-equilibrium electrochemical process at high rates. On the other hand, at low rates (0.5 and 1 A g^−1^), parasitic reaction from gas evolution, possibly catalysed by Mn_3_O_4_ impurity phase, may occur in near-equilibrium condition and deteriorate the electrode materials.

Mn_5_O_8_ electrode materials show relatively low discharge capacities in the initial cycles, and then the capacities continuously increase before showing steady fades for the rest of the cycles. Although similar behaviours have been reported in several electrode systems^8^,^9^,^10^, the exact mechanism of the capacity increase in the initial cycling is still unclear. It has been suggested that initial cycling may help the electrode materials reach their optimal condition by facilitating the ionic transport^9^. We believe our Mn_5_O_8_ electrode may undergo a similar prolonged-conditioning process during the initial cycling, reflected by the overall electrical resistance of the button-cell as calculated by *i*–*R* drop during the discharge processes. Our data show that the cell resistances decrease during the initial cycles and eventually become relatively stable and then start to increase again (Supplementary Fig. 12). Correspondingly, electrode capacities show nearly opposite trends compared with cell resistances: the discharge capacities start to increase at initial cycles in a continuous manor and then show a steady fading for the rest of cycles. We have also pre-soaked the electrode materials in Na_2_SO_4_ electrolyte before cell assembling in order to improve ionic transport at electrode and electrolyte interface. However, it shows that electrode capacities appear very similar trends to those shown in Fig. 3c (Supplementary Figs 10 and 13). The results indicate that Na-ions transport within Mn_5_O_8_ materials during the initial cycling accounts for the large cell resistance.

Figure 3e shows that Mn_5_O_8_ symmetric cells exhibit gravimetric energy and power densities up to 40 Wh kg^−1^ and 17,400 W kg^−1^; and even more impressively, exhibit volumetric energy and power densities of∼13 Wh l^−1^ and 6,000 W l^−1^ (Fig. 3f). These values, obtained after 2,000 cycles, are much higher than those of several aqueous and even non-aqueous devices found in commercial products (less than five cycles) and recent literature in terms of gravimetric performance^11^,^12^,^13^,^14^,^15^, and volumetric performance^16^,^17^,^18^,^19^,^20^. Our Mn_5_O_8_ electrode materials show very competitive performance for aqueous electrochemical energy storage especially at high rates as compared with other Mn-based electrode materials reported previously (Supplementary Table 3). Further increasing the energy density of the Mn_5_O_8_ electrode, especially at low current densities, will be important to develop high-performance aqueous EES devices that could rival their non-aqueous counterparts.

## Discussion

In order to elucidate the mechanisms of this high-voltage and high-rate performance found in the Mn_5_O_8_ system, we first provide synchrotron-based sXAS results with its inherent surface and elemental sensitivities, followed by the DFT calculations.

**Figure 4:**
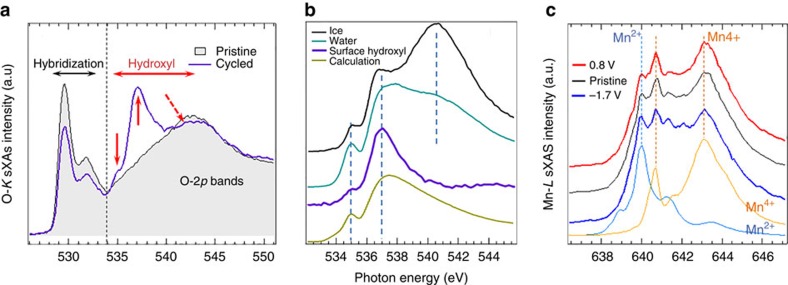
sXAS analysis of surface hydroxylated Mn_5_O_8_. (**a**) O-K sXAS of Mn_5_O_8_ (pristine) and Mn_5_O_8_ electrode after two CV cycles between −1.7 and 0.8 V. Features below 534 eV are from the hybridization of Mn-*3d* and O-*2p* states. The 535 and 537 eV peaks are fingerprints of the water ‘pre-peak' and ‘main peak'. (**b**) A comparison of the O-K sXAS of the surface hydroxyl layer of Mn_5_O_8_ with water^22^, ice^23^ and one of the calculations with aligned H-bonds but lengthened O–O (3.50 and 3.00 Å) distances (Fig. 3a–g in ref. 23). (**c**) Mn *L*-edge of Mn_5_O_8_ (pristine) and Mn_5_O_8_ at different electrochemical cycling stages along with references from MnO (blue) and Li_2_MnO_3_ (yellow).

First, probing the formation of interphase on the surface of Mn_5_O_8_ is not trivial. As most electrode–electrolyte interphases are amorphous, STEM fails to detect the interphase layer (Supplementary Fig. 14). We also carried out neutron PDF analyses on the Mn_5_O_8_ nanoparticles before and after cycling in Na_2_SO_4_ and D_2_O solution. Although Mn_5_O_8_ nanoparticles were prepared in the heavy water (D_2_O) and tested for neutron scattering under an inert gas environment, light water (H_2_O) residuals that might be introduced from moisture in the air showed much strong scattering, so that neutron PDF analysis was unable to identify any interphase on surface of Mn_5_O_8_ materials (Supplementary Fig. 15). Fortunately, surface and elemental sensitivity could be achieved by synchrotron-based soft X-ray spectroscopy through the electron-decay channel^21^. In sharp contrast to neutron scattering, sXAS measurement was conducted in the ultra-high vacuum condition, so that weakly adsorbed water molecules (physically adsorbed or dissociatively adsorbed water) were completely removed and only strongly bonded molecules (for example, interphase) remained on surface. In particular, it has been well established that the hydroxyl group could be probed in water and organic molecules through oxygen K-edge sXAS, with two features at ∼535 and 537 eV from the unsaturated H-bonds, known as the ‘water pre-edge' and ‘water main-peak', respectively^22^,^23^,^24^,^25^. In addition, another post-edge hump above 540 eV is from the saturated H-bonds, especially visible in ice^23^. Figure 4a is the O-K sXAS results collected on the pristine and cycled Mn_5_O_8_ electrodes with a surface probe depth of 10 nm. It is evident that, upon cycling, Mn_5_O_8_ electrode displays both the fingerprinting water-features at the 535 and 537 eV. These features resemble exactly the ‘pre-edge' and ‘main peak' from water. Therefore, the strong spectral lineshape contrast between the pristine and the cycled electrode evidently indicates the formation of hydroxylated species on the electrode surface upon cycling. Moreover, the O–K features between 528 and 534 eV represents the spectroscopic excitations to the hybridized state of O-2*p* and Mn-3*d*, which are split by the crystal field of the local Mn–O coordination geometry^26^. A clear loss of intensity on this hybridization feature is observed after cycling. This intensity loss is further proof of the change of the Mn–O coordination on the surface and is consistent with the formation of hydroxyl group that covers the surface of the electrode.

Due to the great sensitivity of the sXAS lineshape to the hydroxyl structure, the O–K spectra reveals a very interesting molecular arrangement of the hydroxyl groups on the surface of Mn_5_O_8_ electrode. Because the Mn*-*3*d*/O*-*2*p* hybridization features between 528 and 534 eV are purely from Mn_5_O_8_ contribution, we can normalize the spectral intensity to these features and subtract the Mn_5_O_8_ (pristine) signal from the after-cycling data to obtain the surface hydroxyl signal. The spectrum of the obtained surface hydroxyl signal is plotted in Fig. 4b (purple). It is clear that the surface hydroxyl layer is different from ice, missing the high energy hump from the saturated H-bonds in ice^23^. It reproduces the ‘pre-peak' and ‘main peak' of liquid water^22^,^23^, with the ‘main peak' at 537.5 eV dominating the spectrum. The enhancement of this main peak has been predicted by theoretical calculations^23^, which stems from a combination of an extended O–O distance and perfectly aligned H-bonds along the O–O direction. The calculation result of the O–O distance of 3.50 Å with such a well-aligned H-bond is shown in Fig. 4b for comparison. Therefore, the O*–*K sXAS not only evidently shows that a hydroxyl layer is formed on the surface of the Mn_5_O_8_ electrodes after cycling, it also indicates a striking ordering of the absorbed molecules, with perfectly aligned H-bonds along O–O direction, like that in ice, but with a much longer distance between the O atoms. Although the underlying mechanism of the formation of hydroxylated interphase is still unclear, we believe it is largely due to the interaction between Mn_5_O_8_ surface and water during the electrochemical cycling since no hydroxylated interphase is observed in the pristine Mn_5_O_8_ material (Fig. 4a). DFT calculations show that Mn^2+^ terminated (100) surface of Mn_5_O_8_ has a lower surface energy (0.87 J m^−2^) than Mn^4+^ terminated (100) surface (1.80 J m^−2^). Therefore, the interaction between Mn^2+^ and water during the electrochemical cycling might account for the formation of hydroxylated interphase on the Mn^2+^ terminated (100) surface of Mn_5_O_8_.

Second, the charge-storage mechanism is revealed by the Mn L-edge sXAS measurements (Fig. 4c). Mn-L sXAS is a direct probe of the Mn-3*d* electron states, so the spectral lineshape is very sensitive to the normal valence of Mn^27^,^28^. Figure 4c shows the Mn L-edge sXAS data collected on the Mn_5_O_8_ and the reference samples with Mn^2+^ (MnO) and Mn^4+^ (Li_2_MnO_3_) valences. The spectra show that the Mn_5_O_8_ electrode material is composed of Mn^4+^ (641 and 643.5 eV), Mn^2+^ (640 eV) and a trace amount of Mn^3+^, supporting the coexistence of Mn_5_O_8_ phase and trace Mn_3_O_4_ phases. The changes of peak intensity at 640 eV (Mn^2+^), 641 eV (Mn^4+^) and 643.5 eV (Mn^4+^) fingerprints the evolution of oxidation states of Mn with cycling. The Mn_5_O_8_ electrode at a reduced state (−1.7 V) displays a high-intensity ratio of Mn^2+^ to Mn^4+^, while this ratio decreases at the oxidized state (0.8 V), suggesting the transition from Mn^2+^ to Mn^4+^ during oxidation processes. It is therefore evident that a two-charge transfer reaction via Mn^2+^/Mn^4+^ redox couple can be achieved in the layered Mn_5_O_8_ system, indicating a great potential for high-capacity energy storage.

**Figure 5:**
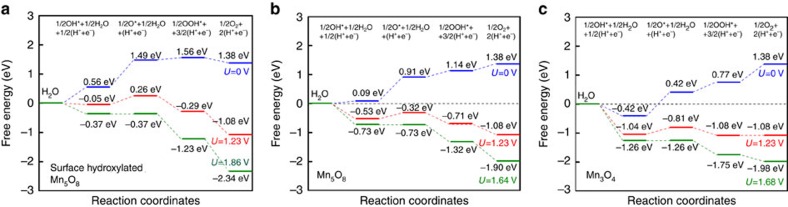
DFT calculations. The free energy evolution for water splitting steps at different potentials on surfaces of (**a**) surface hydroxylated Mn_5_O_8_, (**b**) pure Mn_5_O_8_ and (**c**) Mn_3_O_4_ predicted from DFT.

Furthermore, the mechanistic understanding of the resistive capability of surface-hydroxylated Mn_5_O_8_ towards HER and OER is obtained through a comparative DFT calculation on the surfaces of hydroxylated Mn_5_O_8_, pure Mn_5_O_8_ and Mn_3_O_4_. The Mn^2+^ terminated (100) surface of Mn_5_O_8_ is considered for the study, since our calculation predicted that Mn^2+^ terminated (100) surface had a lower surface energy (0.87 J m^−2^) than Mn^4+^ terminated (100) surface (1.80 J m^−2^). Figure 5 compares water splitting at various potential windows. Nearly all the water splitting steps are energetically unfavourable at zero potential; at a potential of 1.23 V (equilibrium potential of water splitting), all reaction steps are exothermic on three surfaces, except for OH dissociation on hydroxylated Mn_5_O_8_. The limiting potential at which all reaction steps become exothermic is determined to be 1.86, 1.64 and 1.68 V for hydroxylated Mn_5_O_8_, Mn_5_O_8_ and Mn_3_O_4_, respectively, indicating hydroxylated interphase increases the energy barriers for water splitting. The activation energy of each intermediate reaction steps during the HER and OER on the three surfaces was showed (Supplementary Fig. 16). The activation energy of the rate-determining step is all substantially higher for OER, indicating that OER is intrinsically difficult on all three surfaces. The activation energy of water dissociation, limiting step of HER, follows the order of hydroxylated Mn_5_O_8_ (1.41 eV)>Mn_5_O_8_ (0.45 eV)>Mn_3_O_4_ (∼0 eV). Hydroxylated Mn_5_O_8_ possesses an activation energy ∼1 eV higher than that of Mn_3_O_4_, implying that the reaction rate at room temperature (300 K) is at least 17 orders of magnitude slower according to the Arrhenius equation. Therefore, the DFT calculations directly support the experimental observation of high overpotential (>0.6 V) towards HER and OER for the hydroxylated Mn_5_O_8_. It was recently reported that Mn_5_O_8_ nanoparticles showed very good OER activity in a neutral electrolyte in the presence of Mn^3+^ species^29^. Further studies will be needed to elucidate the influence of Mn valences, such as Mn^3+^ (from Mn_3_O_4_ or Mn_2_O_3_ phase) or Mn^2+^/Mn^4+^ (from Mn_5_O_8_), on the gas evolution reactions.

This work shows that an unprecedented potential window of 3.0 V in aqueous Na-ion system can be achieved through the use of surface hydroxylated Mn_5_O_8_ electrodes. Gas evolution reactions are effectively inhibited through the interplay between the Mn^2+^ terminated surface and hydroxylated interphase. In addition, the superior power performance of Mn_5_O_8_ cell can be attributed to the unique layered, bivalence structure of Mn_5_O_8_ that enables facile Na-ion transport. In general, the ion transport through the electrode and electrolyte interface is critical for the rate performance of energy storage devices. Typical approaches to mitigate the transport barrier include decreasing the dimension of electrode materials to nanoscale, designing functional layered structure for facile ion intercalation, coating secondary conductive material on electrode surface, or developing cation-disordered electrode materials with decreased energy barrier for ion transport^6^,^8^,^30^,^31^,^32^. Mn^2+^ terminated (100) surface of Mn_5_O_8_ has plenty of unoccupied sites, and hence provides natural tunnels for Na-ion transport along <100> direction (black arrows) (Supplementary Fig. 17). Through the surface-sensitive soft X-ray spectroscopy, we also reveal the formation of a strikingly ordered ice-like surface hydroxyl layer but with much longer O–O distance after cycling. It is also possible that such a hydroxylated coating on the surface of Mn_5_O_8_ modifies the interaction between Na-ion with Mn_5_O_8_ by facilitating the electrostatic interaction between Na-ion and hydroxyl oxygen (red arrow). Although the electrochemical properties of Mn_5_O_8_ have been largely overlooked since its structure was determined in 1965 (ref. 5), our result shows that Mn_5_O_8_, the only bivalence layered manganese oxide, may serve as a new generation of pseudocapacitor electrode materials for enabling a stable potential window of 3.0 V for aqueous energy storage. Our results may offer a new paradigm for developing electrode materials with a wide potential window and facile charge transfer for aqueous energy storage devices, which exhibits the performance comparable to that of non-aqueous Li-ion batteries, but much safer, less costly and more environmentally benign. In addition, it will be an interesting topic for further studies on whether and how a well ordered hydroxylated interphase could form on the surface of other metal oxides, increase their overpotential towards gas evolution reactions, and therefore increase the potential window of aqueous energy storage.

## Methods

### Materials synthesis

Synthesis was conducted in a magnetically-stirred 50 ml reaction batch of 25.3 mM MnCl_2_·4H_2_O (Alfa Aesar, 99% metals basis) dissolved in 14 ml deionized water (DI H_2_O) (18.2 MΩ; Millipore, Inc.) at room temperature under an open environment. A 20 ml syringe was used to controllably inject a solution of 0.3 M NaOH (Alfa Aesar, 99.99% metals basis) into MnCl_2_ solution at a constant flow rate of 0.167 ml min^−1^ for 50 min using a computer-controlled syringe pump (New Era Syringe Pumps, Inc.). The resulting dark-brown precipitate was permitted to ripen for an additional 30 min at room temperature with stirring. The product was then washed thoroughly with DI H_2_O and ethanol, vacuum-dried and then heated at 270 °C for 2 h.

### Electrochemical tests

The anodic electrochemical properties of the Mn_5_O_8_ product were studied via a three-electrode half-cell, which contained a rotating glassy carbon electrode (Pine Instrument Company) as the working electrode, a platinum wire counter electrode and mercury sulfate (saturated K_2_SO_4_) reference electrode (MSE). The electrochemical half-cell was powered by single-channel electrochemical workstation (Model 600D, CH Instruments, Inc.). The electrode material was prepared by grinding 7 mg Mn_5_O_8_ active material with 3 mg carbon black (Alfa Aesar, 99.9%) and then dissolving the mixture in DI H_2_O to a concentration of 0.5 mg ml^−1^. The resulted solution was subsequently sonicated until manganese oxide was evenly dispersed in the solution and 10 μl suspension taken by pipette (Eppendorf, Inc.) was drop-cast onto the glassy carbon working electrode (0.5 cm in diameter and 0.196 cm^2^ in geometric surface area). Afterwards, the working electrode was dried in vacuum with a loading of Mn_5_O_8_ (3.5 μg) and carbon black (1.5 μg). Electrochemical tests were conducted in a 250 ml flat-bottom flask that contained 100 ml 0.1 M Na_2_SO_4_ electrolyte (Alfa Aesar, 99.9955%) in argon-purged H_2_O with a rotating rate of 500 r.p.m. CV was conducted at voltage scan rates from 1 to 1,000 mV s^−1^ for six segments (three cycles) within a potential range of −1.7 to 0.8 V (versus MSE).

Symmetric two-electrode button-cells were fabricated for long-term durability and stability analyses. Each electrode for full cell tests consisted of a disk (1.5 cm in diameter, 1.77 cm^2^ in area) of Toray carbon paper (E-Tek, Inc.), which was coated with ∼5 mg Mn_5_O_8_ and 1.25 mg carbon black through drop casting. The opposing electrodes were separated via cellulose-based porous filter paper (Whatman). And each button-cell contains 200 μl 1 M Na_2_SO_4_ electrolyte (Alfa Aesar, 99.9955%). A stainless steel plate was placed on one side of the cell and in conjunction with a tightening bolt to ensure good electrical contact. Chronopotentiometry measurements were conducted at constant current densities from 0.5 to 50 A g^−1^ (with respective to each electrode for full cell measurements).

### Data availability

The data that support the findings of this study are available from the corresponding author upon request.

## Additional information

**How to cite this article:** Shan, X. *et al*. Bivalence Mn_5_O_8_ with hydroxylated interphase for high-voltage aqueous sodium-ion storage. *Nat. Commun.* **7,** 13370 doi: 10.1038/ncomms13370 (2016).

**Publisher's note:** Springer Nature remains neutral with regard to jurisdictional claims in published maps and institutional affiliations.

## Supplementary Material

Supplementary Information Supplementary Figures 1-17, Supplementary Tables 1-3, Supplementary Notes 1-2, Supplementary Methods and Supplementary References
