# Supplementary material for: Bivalence Mn5O8 with hydroxylated interphase for high-voltage aqueous sodium-ion storage
Source: Nat Commun. 2016 Nov 15;7:13370. doi: 10.1038/ncomms13370 (PMC5116065; doi:10.1038/ncomms13370)
Supplement: Supplementary Information — Supplementary Figures 1-17, Supplementary Tables 1-3, Supplementary Notes 1-2, Supplementary Methods and Supplementary References [file ncomms13370-s1.pdf]

Supplementary Information

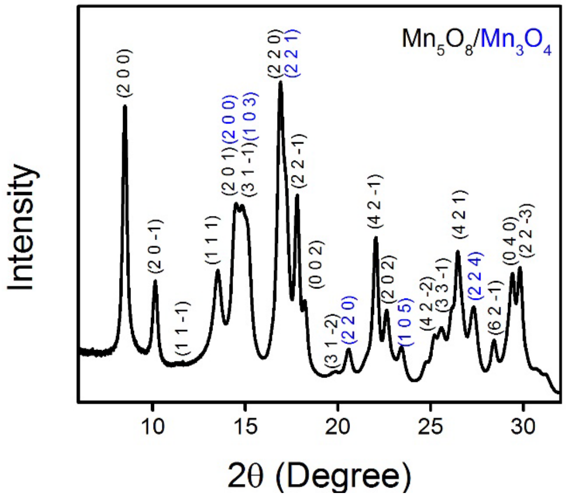

Supplementary Figure 1 | XRD characterization. XRD pattern of Mn_5O_8 nanoparticles (black) and Mn_3O_4 (blue) impurity phase.

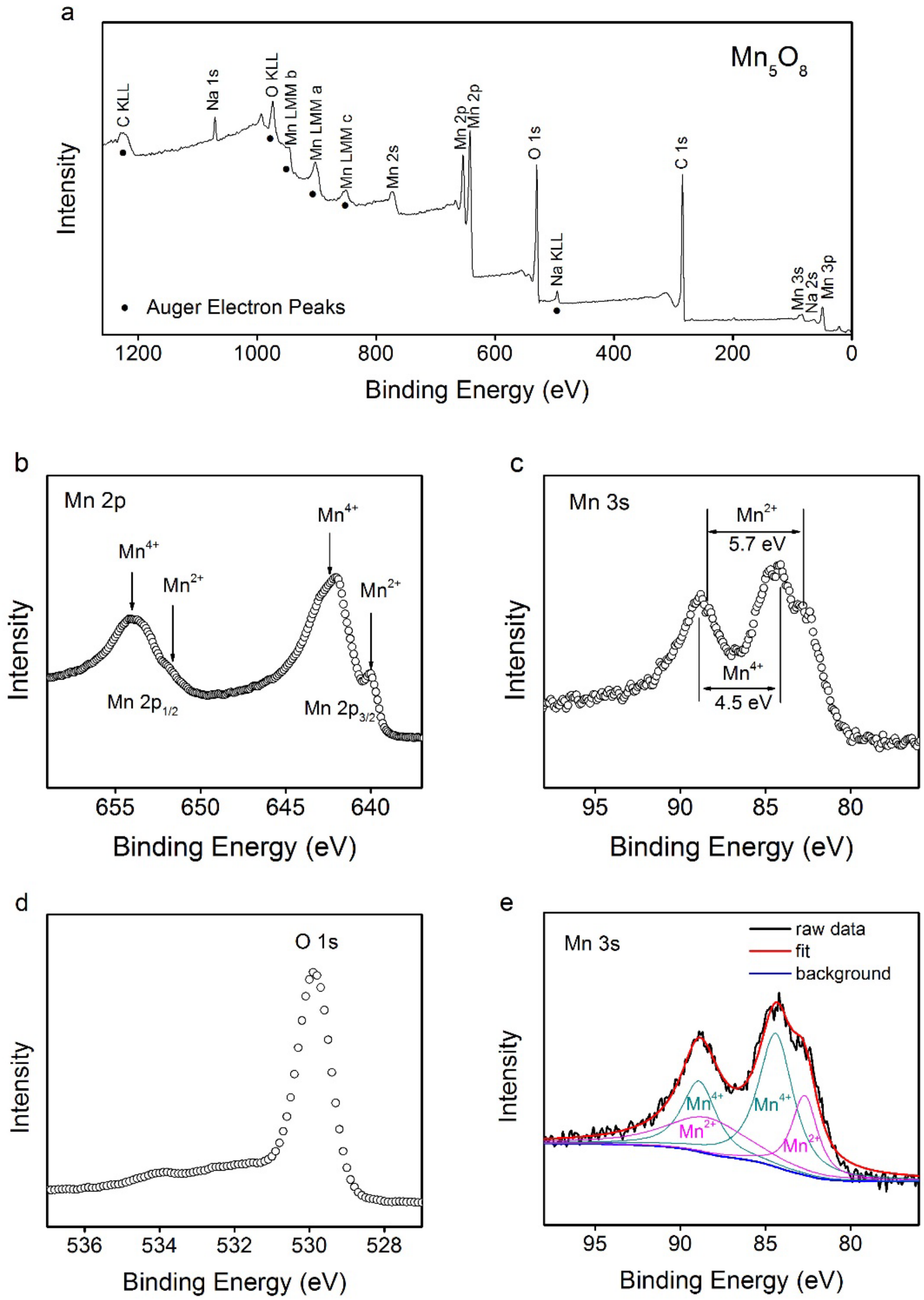

Supplementary Figure 2 | XPS characterization of Mn_5O_8 nanoparticles. (**a**) XPS survey spectrum, (**b**) Mn 2p, (**c**) Mn 3s and (**d**) O 1s chemical states of Mn_5O_8 nanoparticles. (**e**) The fitting of Mn 3s spectra shows the Mn^{2+} and Mn^{4+} with an integrated intensity ratio of 1:2.

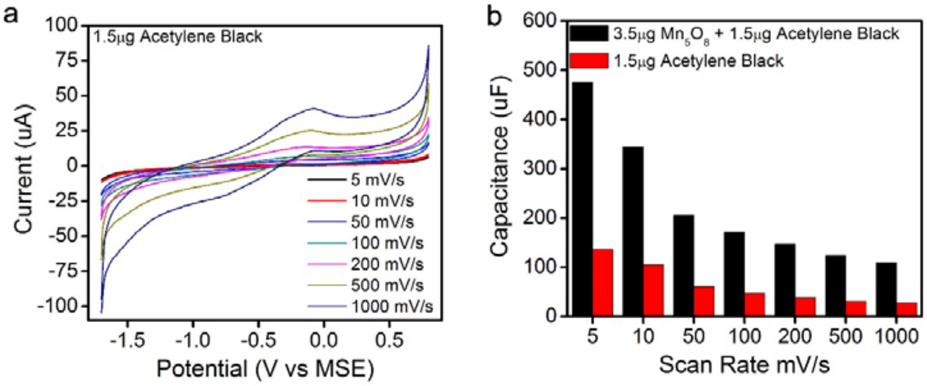

Supplementary Figure 3 | Electrochemical half-cell measurements of carbon black. (**a**) The CVs of carbon black, and (**b**) calculated capacitance of carbon black showing that the Mn_5O_8 materials contributed to the majority of the capacitance in the mixture of Mn_5O_8 and carbon black.

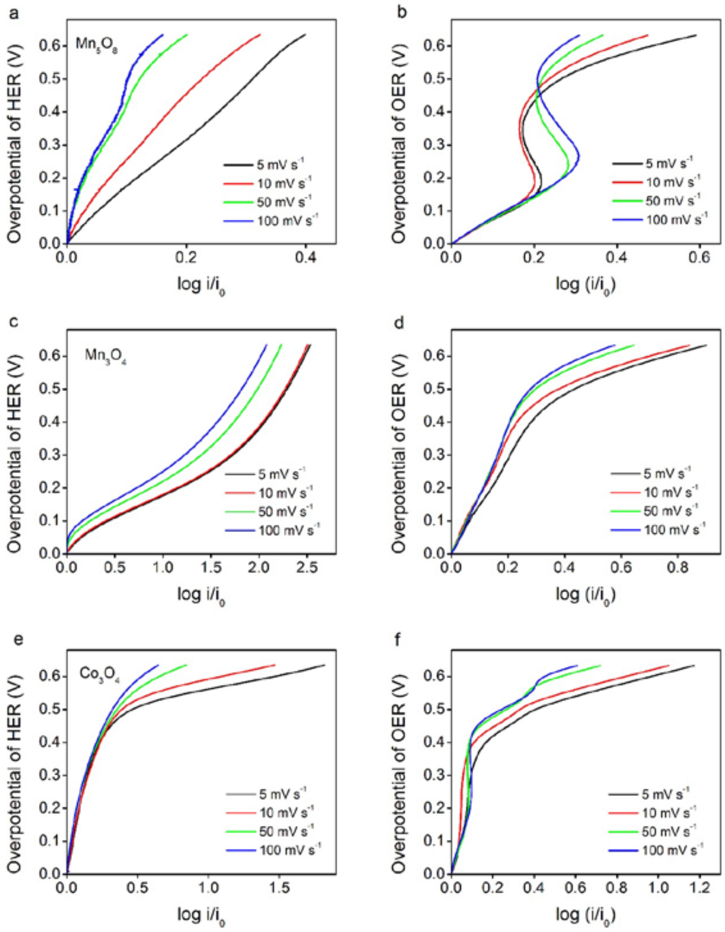

Supplementary Figure 4 | TAFEL plots of HER and OER. (**a, b**) Mn_5O_8 nanoparticles, and commercial (**c, d**) Mn_3O_4, and (**e, f**) Co_3O_4 nanopowders. The measurements were carried using a three-electrode half-cell in 0.1M Na_2SO_4 electrolyte with a rotating disc working electrode (500 rpm).

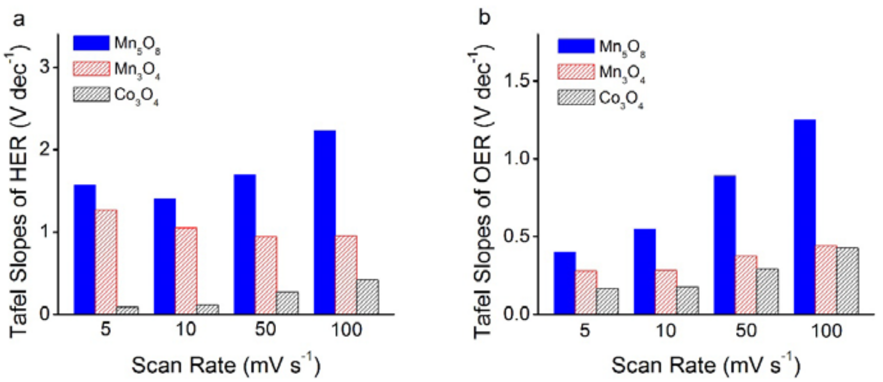

Supplementary Figure 5 | TAFEL slopes analysis. TAFEL slopes of (**a**) HER and (**b**) OER as function of scan rates for Mn_5O_8 nanoparticles and commercial Mn_3O_4 and Co_3O_4 nanopowders.

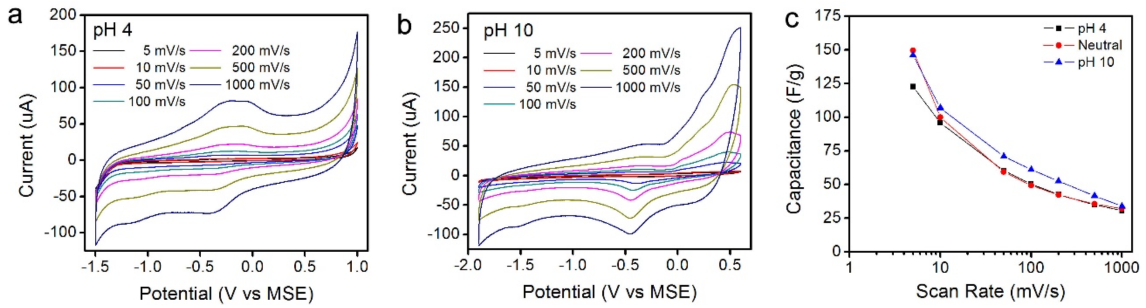

Supplementary Figure 6 | Electrochemical half-cell studies at various pH values. (**a, b**) CVs of 3.5 ug Mn_5O_8 and 1.5 ug carbon black measured at pH = 4 and pH = 10, and (**c**) calculated capacitance at various pHs.

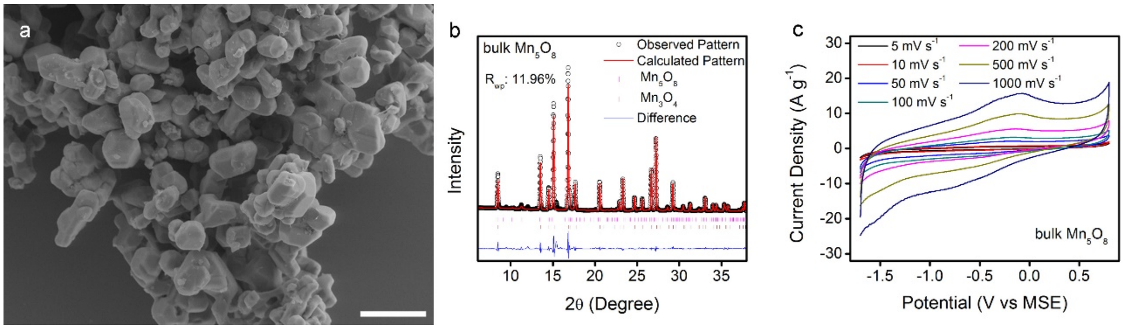

Supplementary Figure 7 | Structural and electrochemical half-cell studies of bulk Mn_5O_8. (**a**) Scanning electron microscopy (SEM) image, (**b**) XRD spectra, and (**c**) CVs of bulk Mn_5O_8 materials with an average diameter of 2 µm. Scale bars, 5 µm (**a**).

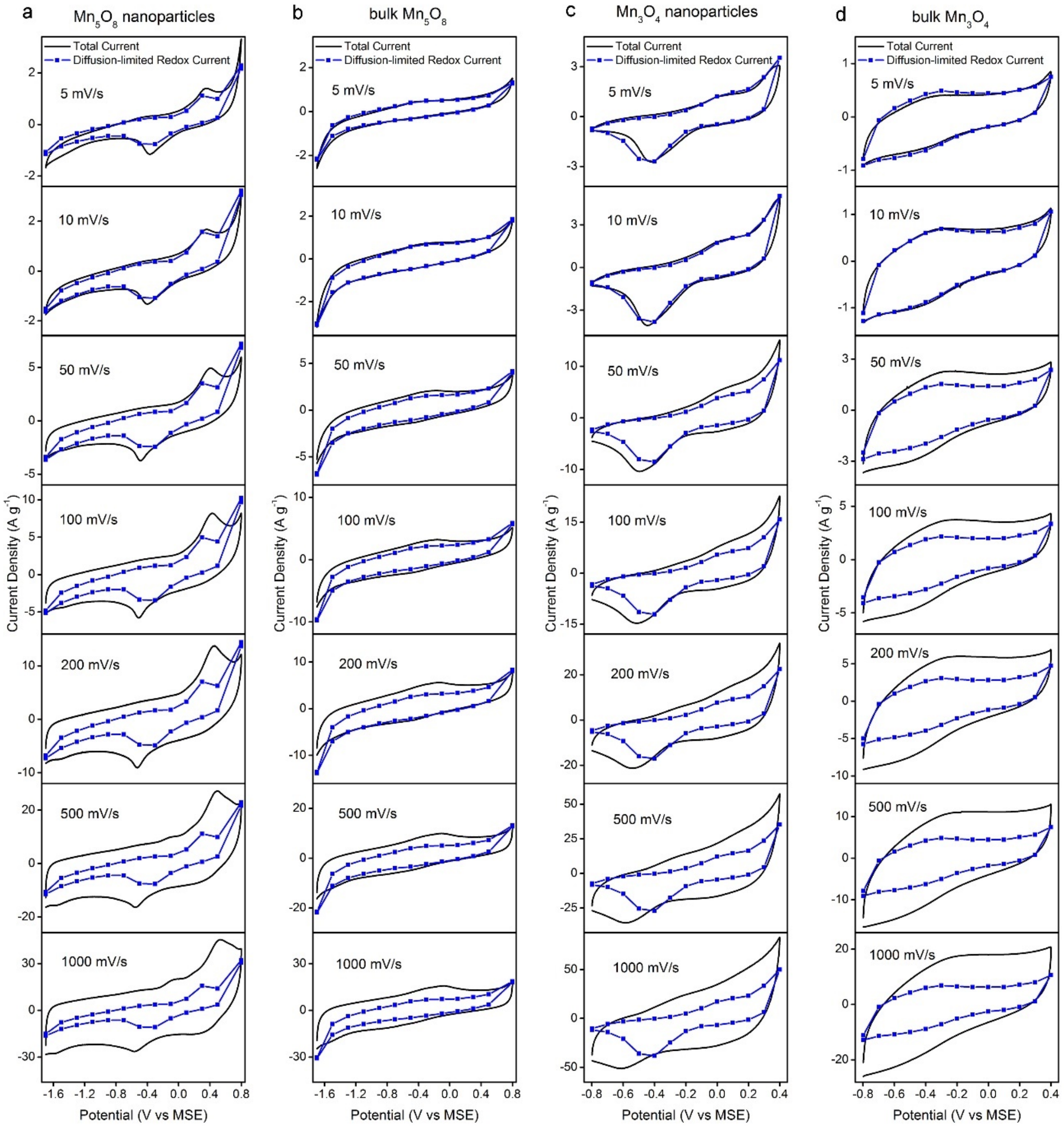

Supplementary Figure 8 | Electro-kinetics analysis. Electro-kinetics studies of (**a**) Mn_5O_8 nanoparticles, (**b**) bulk Mn_5O_8, (**c**) Mn_3O_4 nanoparticles and (**d**) Mn_3O_4 bulk materials, showing the ratio between total current and diffusion-limited redox current. The integrated curves provide the contribution from diffusion-limited redox process and capacitive process to the overall charge stored as shown in Fig. 2c.

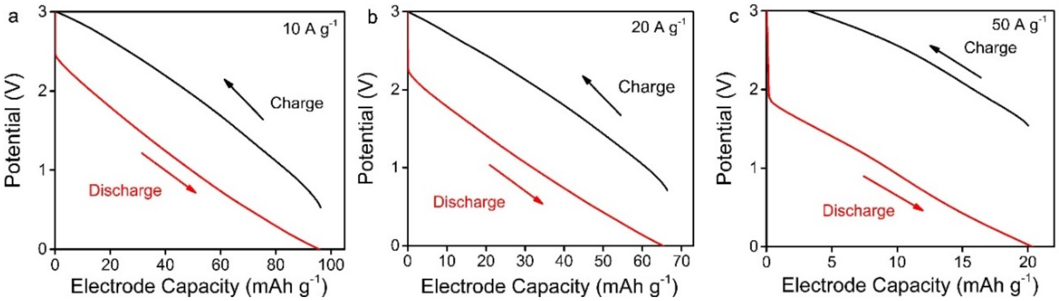

Supplementary Figure 9 | Galvanostatic change/discharge studies. Electrode capacities of Mn_5O_8 during galvanostatic charging and discharging at current densities of (**a**)10 A g^{-1}, (**b**) 20 A g^{-1}, and (**c**) 50 A g^{-1}.

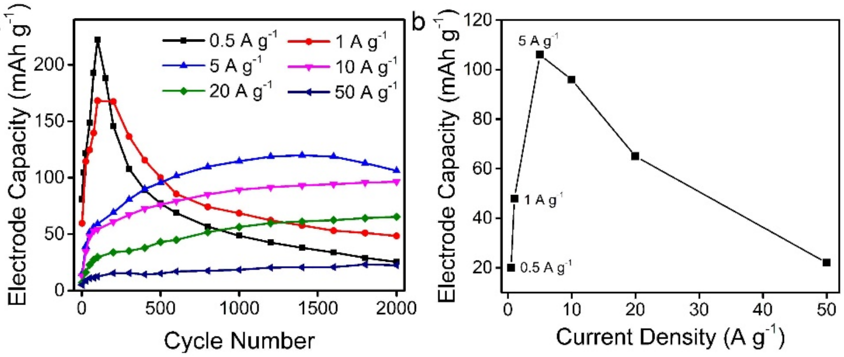

Supplementary Figure 10 | Mn_5O_8 full cell performance at low/high current densities. (**a**) Electrode capacities as a function of cycling number of Mn_5O_8 cells at current densities from 0.5 to 50 A g^{-1}; (**b**) electrode capacities at current densities from 0.5 to 50 A g^{-1} after 2,000 cycles.

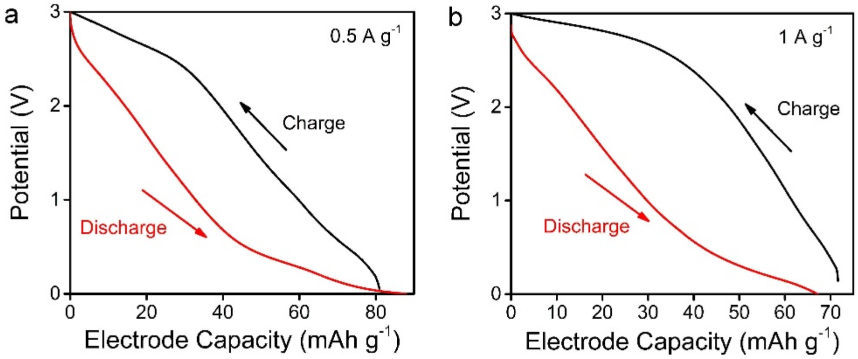

Supplementary Figure 11 | Galvanostatic charge/discharge studies at low current densities. Electrode capacities of Mn_5O_8 during galvanostatic charging and discharging at (**a**) 0.5 A g^{-1} and (**b**) 1 A g^{-1} (2nd cycle data).

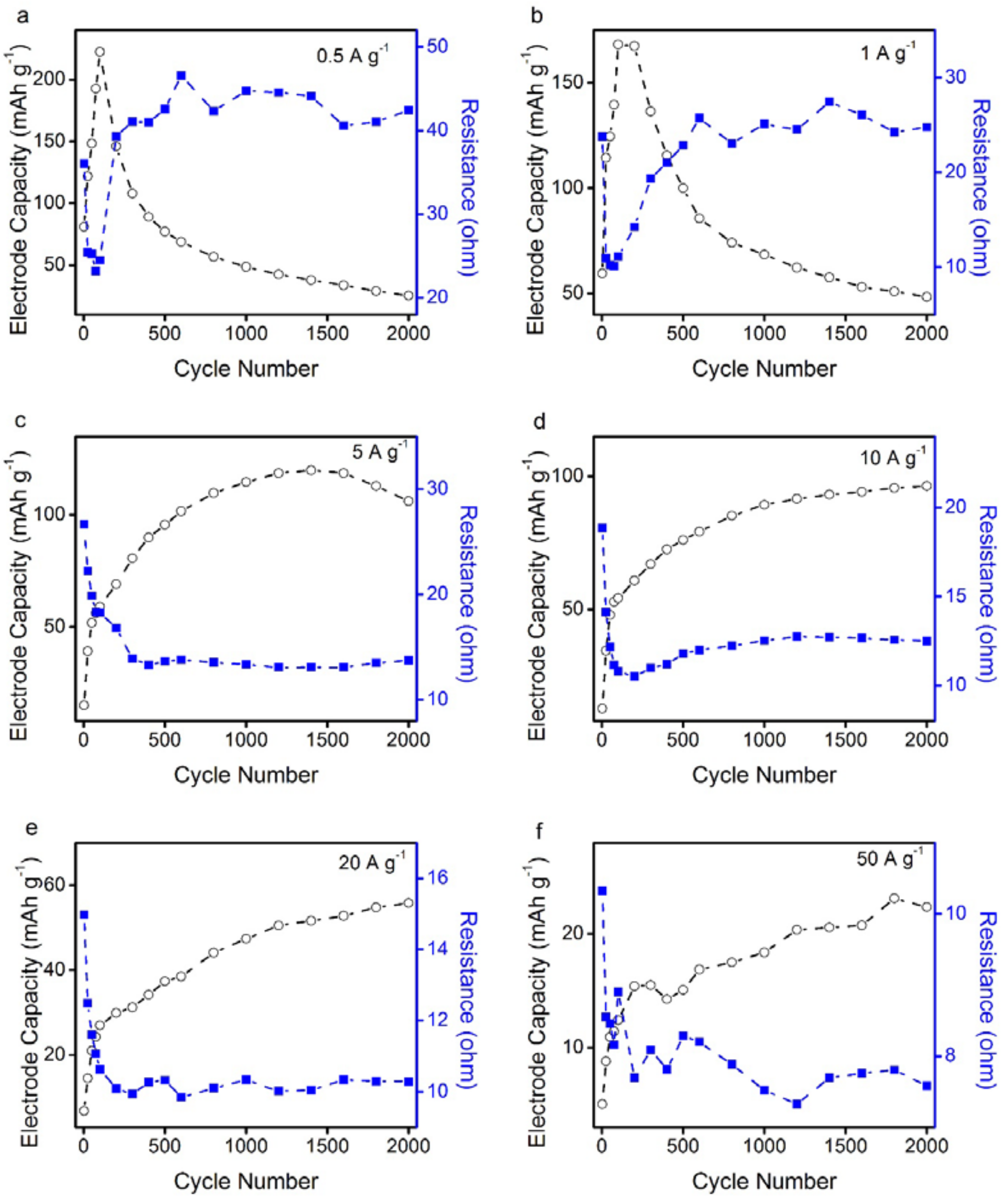

Supplementary Figure 12 | Electrode capacities and cell resistances analysis. Electrode capacities and electrical resistances of Mn_5O_8full cell calculated from i-R drop during the discharge process as functions of cycle number at current densities of (**a**) 0.5 A g^{-1}, (**b**) 1 A g^{-1}, (**c**) 5 A g^{-1}, (**d**) 10 A g^{-1}, (**e**) 20 A g^{-1} and (**f**) 50 A g^{-1}.

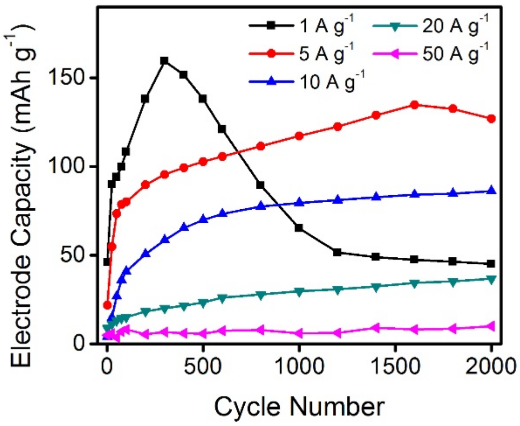

Supplementary Figure 13 | Pre-soaked Mn_5O_8 full cell performance. Electrode capacities as a function of cycle number for Mn_5O_8 cells at current densities from 0.5 to 50 A g^{-1}. The electrode materials are pre-soaked in the electrolyte before assembling.

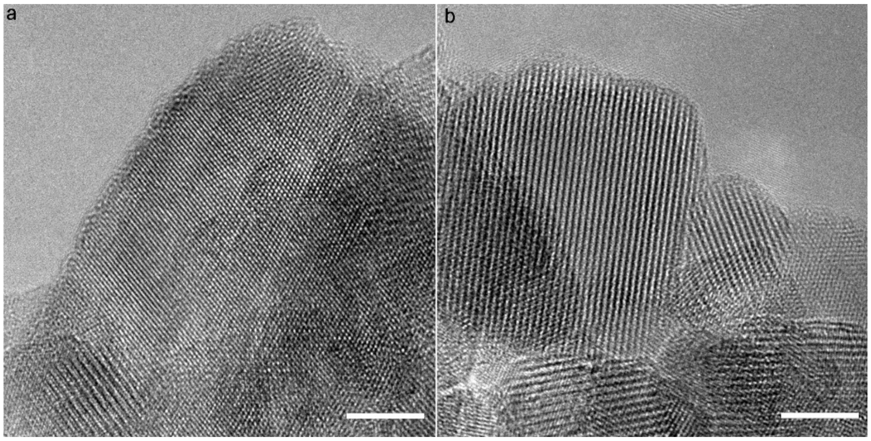

Supplementary Figure 14 | STEM characterizations. STEM of Mn_5O_8 nanoparticles (**a**) before and (**b**) after cycling. No hydroxylated coating is discernable on surface after cycling. Scale bars, 5 nm (**a, b**).

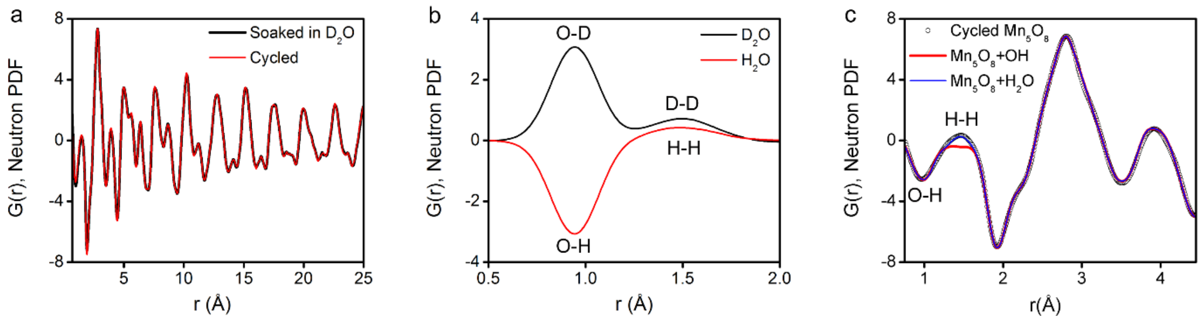

Supplementary Figure 15 | Neutron Scattering. (a) Neutron PDF of the Mn_5O_8 material soaked in D_2O and the Mn_5O_8 cycled ex-situ in Na_2SO_4 (light water). The PDFs showed little difference, notably both PDFs containing a strong negative peak at ~0.98 Å. (b) Simulated PDFs of a single D_2O and H_2O molecule, exhibiting the difference between water molecules with different isotopes of hydrogen. Because the neutron scattering length of Deuterium is positive the O-D correlation is positive, whereas the neutron scattering length of Hydrogen is negative the O-H correlation is negative. Both the D-D and H-H correlations of both the light and heavy water are positive. As the peak at ~0.98 Å in the PDF of both cycled and soaked Mn_5O_8 data is strongly negative, the D_2O substitution was unsuccessful, and is therefore the signal is due to light water in the sample. (c) Models containing Mn_5O_8 + O-H species and Mn_5O_8 + H_2O molecule were fit to the data of the cycle Mn_5O_8. The model containing O-H species was unable to fit the peak at ~1.5 Å, but the model with water was having the H-H correlation that the Mn_5O_8 + O-H model lacks.

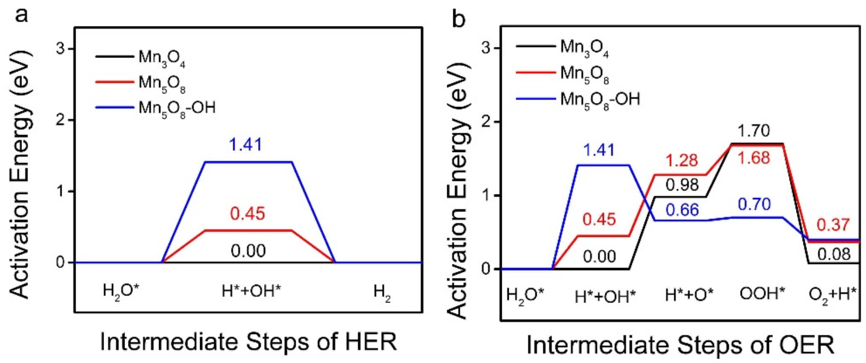

Supplementary Figure 16 | Activation energies of HER/OER intermediate steps. The activation energies of intermediate reaction steps of (**a**) HER and (**b**) OER on the (100) surface of surface hydroxylated Mn_5O_8 (Mn_5O_8-OH) or as-made Mn_5O_8 and (101) surface of Mn_3O_4 (the asterisk symbol denotes the chemical species that are adsorbed on the surface).

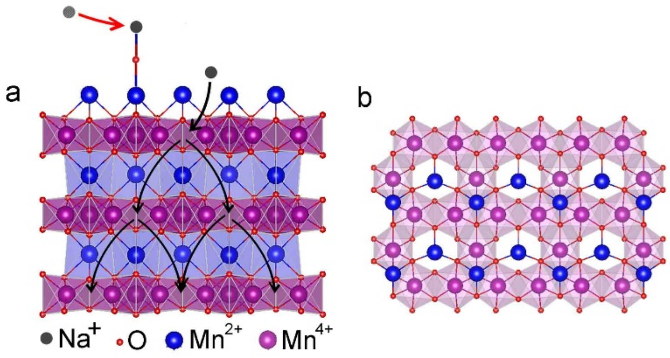

Supplementary Figure 17 | Schematic Na-ion transport through hydroxylated Mn_5O_8. (**a**) Side view along <010> direction and (**b**) top view along <100> direction of Mn_5O_8 stacked lattice demonstrates the alternative arrangement of layered Mn^{4+} (purple) and Mn^{2+} (blue) atoms. Na-ion (grey) transport can be achieved from the pathway along empty sites along <100> direction as marked in black arrows, and can be achieved from electrostatic interaction with the hydroxylated interphase (-OH) which is marked with in a red arrow.

Supplementary Table 1. Structural parameters obtained from fitting of X-ray scattering PDF.

R_{wp}	0.148756					
Lattice Parameters	a	b	c	α	β	γ
	10.4094	5.7259	4.87682	90	109.72	90

Atom	Label	x	y	z	Occupancy	Uiso
Mn	Mn4+	0	0	0.5	0.8559	0.006734
Mn	Mn4+	0	0.257024	0	1	0.006474
Mn	Mn2+	0.280458	0	0.341775	0.8255	0.011694
O	O2-	0.903085	0.246548	0.567454	1	0.173223
O	O2-	0.899653	0	0.091055	1	0.008911
O	O2-	0.361381	0	0.065537	1	0.091072

Supplementary Table 2. Structural parameters obtained from fitting of neutron scattering PDF.

R_{wp}	0.130789					
Lattice Parameters	a	b	c	α	β	γ
	10.4151	5.72684	4.89384	90	110.117	90

Atom	Label	x	y	z	Occupancy	Uiso
Mn	Mn4+	0	0	0.5	0.8559	0.028728
Mn	Mn4+	0	0.257187	0	1	0.055603
Mn	Mn2+	0.246953	0	0.348702	0.8255	0.026482
O	O2-	0.895298	0.230424	0.579538	1	0.022677
O	O2-	0.873524	0	0.051812	1	0.02266
O	O2-	0.406983	0	0.119846	1	0.023713

Supplementary Table 3. Summarized electrochemical performance of various Mn based electrode materials for aqueous electrochemical energy storage.

Anode	Cathode	Electrolyte	C-rate	Voltage (V)	Electrode Capacity (mAh g^{-1})	Ref.
Mn_5O_8/Carbon Black	Mn_5O_8/Carbon Black	1 M Na_2SO_4	86	3.0	106	Our report
$NaTi_2(PO_4)_3$	$NaMnO_2$	2 M CH_3COONa	1	1.3	132	1
$NaTi_2(PO_4)_3$	$Na_{0.44}MnO_2$	1 M Na_2SO_4	3	1.3	103	2
Graphene	MnO_2/Graphene	1 M Na_2SO_4	7.4	2.0	68	3
Activated Carbon	$LiMn_2O_4$	0.5 M Li_2SO_4	9	1.8	110	4
TiO_2	$LiMn_2O_4$	3.5 M LiCl/ 0.25 M Li_2SO_4	10	2.0	135	5
Activated Carbon	$Na_{0.44}MnO_2$	1 M Na_2SO_4	0.7	1.2	35	6
Activated Carbon	MnO_2	0.1 M K_2SO_4	29	2.0	51	7
FeO_x/Carbon	MnO_2/Carbon	2.5 M Li_2SO_4	7.6	1.8	66	8

Supplementary Note 1. Capacitanceand electro-kinetic analyses in half-cell.

The CV measurements were conducted in 0.1M Na_2SO_4 electrolyte at scans rates from 1 to 1000 mV s^{-1} within a potential range from – 1.7 V to 0.8 V (vs. MSE).

The mass-specific capacitance (C_{MS}) from CV measurement in half-cell was calculated from:

$$C_{MS} = \frac{i}{(dV/dt)*m} = \int_{t_0}^{t_F} \frac{i}{\Delta V*m} dt \qquad \text{Supplementary Eq. (1)}$$

Where i (A) is the measured current at certain time of t (s), m (g) is the mass of active material loaded on working electrode, ΔV (V) is potential window, t_0 (s)and t_F (s) are respective times at the initial potential and the final potential.

The current contributed from surface-controlled capacitive process can be represented by:

$$i_1 = k_1 v \qquad \text{Supplementary Eq. (2)}$$

Where i is the current (A) and v is the scan rate (mV s^{-1}).

While the current contributed from diffusion-limited redox process can be represented by:

$$i_2 = k_2 v^{1/2} \qquad \text{Supplementary Eq. (3)}$$

Therefore, the overall current can be represented by:

$$i = k_1 v + k_2 v^{1/2} \qquad \text{Supplementary Eq. (4)}$$

After rearrangement, it can be written as:

$$\frac{i}{v^{1/2}} = k_1 v^{1/2} + k_2 \qquad \text{Supplementary Eq. (5)}$$

By plotting $i/v^{½}$ vs. $v^{½}$ curves at a given potential, k_1 and k_2 values can be determined, and hence the contribution of capacitive charge and diffusion-limited redox charge during the CV measurements in half-cell can be analyzed quantitatively.

TAFEL analyses of HER and OER were conducted in half-cell. The TAFEL equation relates the HER and OER activities to their overpotentials (η) and is expressed as:

$$\eta = A \times \log(\frac{i}{i_0}) \qquad \text{Supplementary Eq. (6)}$$

where i is experimentally observed current density, i_0 is the exchange current density (the equilibrium current density when η = 0) and A is the TAFEL slope. The TAFEL slope is calculated from the liner region of TAFEL plots (η vs. log i/i_0), typically in the overpotential range from ~ 0.5 V to ~ 0.63 V.

Supplementary Note 2. Electrochemical full cell calculations.

The cell capacitance C_{cell} (F), cell mass-specific capacitance C_{MS} (F g^{-1}), electrode mass-specific capacitance $C_{MS(electrode)}$ (F g^{-1}), discharge cell capacity by mass $C_{discharge}$ (mAh g^{-1}), discharge electrode capacity by mass $C_{discharge(electrode)}$ (mAh g^{-1}), specific energy E_{MS} (Wh kg^{-1}), specific power P_{MS} (W kg^{-1}) and coulombic efficiency (η) and energy efficiency (γ) were calculated.

Cell capacitance: $C_{cell} = \frac{it}{U}$ Supplementary Eq. (7)

Cell mass-specific capacitance: $C_{MS} = \frac{it}{U*M} = \frac{it}{U*2m}$ Supplementary Eq. (8)

Electrode mass-specific capacitance: $C_{MS(electrode)} = 4C_{MS} = \frac{2it}{U*M/2} = \frac{2it}{U*m}$

Supplementary Eq. (9)

Discharge cell capacity by mass: $C_{discharge} = \frac{it}{3.6*M} = \frac{it}{3.6*2m}$ Supplementary Eq. (10)

Discharge electrode capacity by mass:

$C_{discharge(electrode)} = 4C_{discharge} = \frac{2it}{3.6*M/2} = \frac{2it}{3.6*m}$ Supplementary Eq. (11)

Specific energy: $E_{MS} = \frac{\frac{1}{2}CU^2}{3.6*M} = \frac{\frac{1}{2}*itU}{3.6*M} = \frac{\frac{1}{2}*itU}{3.6*2m}$ Supplementary Eq. (12)

Specific power: $P_{MS} = \frac{3600*E_{MS}}{t}$ Supplementary Eq. (13)

Coloumbic efficiency: $\eta = \frac{Q_{discharge}}{Q_{charge}} = \frac{it_{discharge}}{it_{charge}} = \frac{t_{discharge}}{t_{charge}}$ Supplementary Eq. (14)

Energy efficiency: $\gamma = \frac{\int_{V_{discharge(0)}}^{V_{discharge(F)}} C_{discharge(electrode)} dV}{\int_{V_{charge(0)}}^{V_{charge(F)}} C_{charge(electrode)} dV}$ Supplementary Eq. (15)

Cell resistance: $R = \frac{\Delta V}{i}$ Supplementary Eq. (16)

Where i (A) is the applied constant current, t (s) is discharge time of the cell device, U (V) is potential window, ΔV (V) is the i-R drop during discharge curve, M (g) is the total mass of active materials on both electrodes and m (g) is the mass of active materials on one electrode.

Supplementary Methods:

Structural characterization. High resolution transmission electron microscopy (HRTEM) images were collected using aberration-corrected Hitachi HD 2700C equipped with a modified Gatan Enfina ER spectrometer at the Center for Functional Nanomaterials at the Brookhaven National Laboratory. Regular TEM images were obtained using a Zeiss/LEO 922 Omega TEM at the University of New Hampshire. Powder XRD spectra were obtained at beam line 17-BM (λ = 0.72768 Å) at Advanced Photon Source, Argonne National Laboratory. SEM images were collected by Lyra 3 GMU FIB SEM (Tescan, Inc.) at University of New Hampshire.

Pair distribution function (PDF) analysis of neutron and X-ray scattering. The neutron scattering experiments were conducted at the Nanoscale-Ordered Materials Diffractometer (NOMAD) beamline at Spallation Neutron Source at Oak Ridge National Laboratory, and X-ray scattering were conducted at the 11-ID-B beamline at Advanced Photon Source at Argonne National Laboratory. The PDF analysis was conducted using PDFgui software. The X-ray PDF was fit from 1 Å to 50 Å with using a Q_{max} value of 23.5 $Å^{-1}$ and the Neutron PDF was fit from 1.6 Å to 50 Å using a Q_{max} value of 25 $Å^{-1}$. The refinement of the X-ray and neutron PDFs resulted in lattice parameters that are in good agreement with one another. The PDF analysis also showed that the nanoparticles consisted of 80.8% of Mn_5O_8 and 19.2% of Mn_3O_4 by mass from neutron scattering, and 80.4% of Mn_5O_8 and 19.6% of Mn_3O_4 by mass from X-ray scattering experiments.

Soft X-ray Absorption Spectroscopy (sXAS): sXAS was performed at Beamline 8.0.1 at the Advanced Light Source (ALS) of the Lawrence Berkeley National Lab (LBNL). The undulator and spherical grating monochromator supply a linearly polarized photon beam with resolving power up to 6000. The experimental energy resolution is about 0.15eV. The electrode samples were loaded into the ultra-high vacuum sXAS characterization chamber from an Argon glove box through a sample transfer kit to avoid any air exposure. Experiments were performed at room temperature and with the linear polarization of the incident beam oriented at 45 ^{o} to the electrode surface. The sXAS spectra were collected using total electron yield (TEY) mode with a probing depth of about 10 nm. All the spectra have been normalized to the beam flux that was measured simultaneously by an upstream gold mesh.

Density Functional Theory (DFT) calculations: The reaction free energies and activation energies for hydrogen evolution reaction (HER) and oxygen evolution reaction (OER) on the (100) surface of Mn_5O_8 with and without hydroxylated layer were calculated using the first principles. For the calculation of water splitting at various electrochemical potential windows (Fig. 5), the following steps were considered:

$$\mathrm{H_2O} \overset{a}{\rightarrow} \frac{1}{2}\mathrm{OH}^* + \frac{1}{2}\mathrm{H_2O} + \frac{1}{2}(\mathrm{H}^+ + e) \overset{b}{\rightarrow} \frac{1}{2}\mathrm{O}^* + \frac{1}{2}\mathrm{H_2O} + (\mathrm{H}^+ + e)$$

$$\overset{c}{\rightarrow} \frac{1}{2}\mathrm{OOH}^* + \frac{3}{2}(\mathrm{H}^+ + e) \overset{d}{\rightarrow} \frac{1}{2}\mathrm{O_2} + 2(\mathrm{H}^+ + e) \qquad \text{Supplementary Eq. (17)}$$

The reaction kinetics on the (100) surface of Mn_5O_8 with and without OH adsorption were investigated by calculating the reaction activation energy of each intermediate reaction step

(Supplementary Fig. 16). On all the surfaces, we assume that the reaction mechanism of HER is surface hydrogen association while the reaction mechanism of OER is surface OOH intermediate reaction as shown in Fig. 5a and 5b, respectively. For both HER and OER, the activation energy of water adsorption was ignored since the negative adsorption energy of water -0.87 eV on both surfaces indicates that water can be easily adsorbed onto the surface. We also ignored the activation energy of the combination of surface hydrogen and the dissociation of OOH for these reactions are usually not rate determining.

All the spin-polarised DFT calculations were performed using Vienna Ab-initio Simulation Package (VASP)[9,10]. Project augment wave (PAW) method was used to describe the interaction between the ion and valence electrons[11]. The exchange-correlation functional developed by Perdew, Burke and Ernzerhof (PBE)[12] was used. The energy cutoff of all the calculations was set to be 400 eV as suggested by Franchini *et* al[13]. In addition, PBE+U method following the approach of Dudarev[14] was used to describe the correlation between the d electrons of Mn. The U value was set as 4 eV as suggested by Wang *et al*[15]. Furthermore, the transition state of all the relevant reactions on the surfaces were calculated by climb-image nudged-elastic band (CINEB) method[16], in which all the force components both along and perpendicular to the tangent of the reaction path were relaxed to be less than 0.05 eV $Å^{-1}$.

Supplementary References

1. Hou, Z., Li, X., Liang, J., Zhu, Y. & Qian, Y. An Aqueous Rechargeable Sodium Ion Battery Based on a $NaMnO_2$-$NaTi_2(PO_4)_3$ Hybrid System for Stationary Energy Storage. *J. Mater. Chem. A* **3**, 1400-1404, (2015)
2. Li, Z., Young, D., Xiang, K., Carter, W. C. & Chiang, Y. M. Towards High Power High Energy Aqueous Sodium-Ion Batteries: The $NaTi_2(PO_4)_3/Na_{0.44}MnO_2$ System. *Adv. Energy Mater.* **3**, 290-294, (2013)
3. Wu, Z. S., Ren, W., Wang, D. W., Li, F., Liu, B. & Cheng, H. M. High-Energy MnO_2 Nanowire/Graphene and Graphene Asymmetric Electrochemical Capacitors. *ACS Nano*. **4**, 5835-5842, (2010)
4. Qu, Q., Fu, L., Zhan, X., Samuelis, D., Maier, J., Li, L., Tian, S., Li, Z. & Wu, Y. Porous $LiMn_2O_4$ as A Cathode Material with High Power and Excellent Cycling for Aqueous Rechargeable Lithium Batteries. *Energy Environ. Sci.* **4**, 3985-3990, (2011)
5. Liu, S., Ye, S. H., Li, C. Z., Pan, G. L. & Gao, X. P. Rechargeable Aqueous Lithium-Ion Battery of $TiO_2/LiMn_2O_4$ with a High Voltage. *J. Electrochem. Soc.* **158** (12) A1490-A1497, (2011)
6. Tevar, A. D. & Whitacre, J. F. Relating Synthesis Conditions and Electrochemical Performance for the Sodium Intercalation Compound $Na_4Mn_9O_{18}$ in Aqueous Electrolyte. *J. Electrochem. Soc.* **157** (7) A870-A875, (2010)
7. Brousse, T. & Simon, P. Long-term cycling behavior of asymmetric activated carbon/MnO_2 aqueous electrochemical supercapacitor. *J. Power Sources.* **173** 633-641, (2007)
8. Sassin, M. B., Chervin, C. N., Rolison D. R. & Long, J. F. Redox Deposition of Nanoscale Metal Oxides on Carbon for Next-Generation Electrochemical Capacitors. *Accounts of Chemical Research* **46** (5) 1062-1074, (2013)
9. Kresse, G. & Hafner, J. Ab initio molecular dynamics for liquid metals. *Phys. Rev. B* **47**, 558, (1993)
10. Kresse, G. & Furthmuller, J. Efficiency of *ab-initio* total energy calculations for metals and semiconductors using a plane-wave set. *Comp. Mat. Sci.* **6**, 15-50, (1996)
11. Kresse, G. & Joubert, D. From ultrasoft pseudopotentials to the projector augmented-wave method. *Phys. Rev. B* **59**, 1758, (1999)
12. Perdew, J. P., Burke, K. & Ernzerhof, M. Generalized gradient approximation made simple. *Phys. Rev. Lett.* **77**, 3865, (1996)

13. Franchini, C., Podloucky, R., Paier, J., Marsman, M. & Kresse, G. Ground-state properties of multivalent manganese oxides: Density functional and hybrid density functional calculations. *Phys. Rev. B* **75**, 195128, (2007)
14. Dudarev, S. L., Botton, G. A., Savrasov, S. Y., Humphreys, C. J. & Sutton, A. P. Electron-energy-loss spectra and the structural stability of nickel oxide: An LSDA+U study. *Phys. Rev. B* **57,** 1505, (1998)
15. Wang, L., Maxisch, T. & Ceder, G. Oxidation energies of transition metal oxides within the GGA+U framework. *Phys. Rev. B* **73**, 195107, (2006)
16. Henkelman, G., Uberuaga B. P. & Jónsson, H. A climbing image nudged elastic band method for finding saddle points and minimum energy paths. *J. Chem. Phys.* **113**, 9901, (2000)
